# Cryptotanshinone possesses therapeutic effects on ischaemic stroke through regulating STAT5 in a rat model

**DOI:** 10.1080/13880209.2021.1914672

**Published:** 2021-04-29

**Authors:** Feihong Zhu, Hehe Chen, Meifei Xu, Xiajun Zhang, Jing Yu, Yali Pan, Weixin Zhu

**Affiliations:** Department of Rehabilitation, Jinhua Central Hospital, Jinhua City, P.R. China

**Keywords:** Tregs, FOXP3, CD4 ^+^ CD25 ^+^ FOXP3^+^ Treg cells

## Abstract

**Context:**

Cryptotanshinone (CT), a lipophilic compound extracted from roots of *Salvia miltiorrhiza* Bunge (Lamiaceae) (Danshen), has multiple properties in diseases, such as pulmonary fibrosis, lung cancer, and osteoarthritis. Our previous findings suggest that CT plays a protective role in cerebral stroke. However, the molecular mechanisms underlying CT protection in ischaemic stroke remain unclear.

**Objective:**

This study examines the effect of CT on ischaemic stroke.

**Materials and methods:**

We used the middle cerebral artery occlusion (MCAO) rat (Sprague-Dawley rats, 200 ± 20 g, *n* = 5) model with a sham operation group was treated as negative control. MCAO rats were treated with 15 mg/kg CT using intragastric administration. Moreover, TGF-β (5 ng/mL) was used to treat MCAO rats as a positive control group.

**Results:**

The 50% inhibitory concentration (IC_50_) of CT on CD4^+^ cell damage was 485.1 μg/mL, and median effective concentration (EC_50_) was 485.1 μg/mL. CT attenuates the infarct region in the MCAO model. The percentage of CD4^+^CD25^+^FOXP3^+^ Treg cells in the peripheral blood of the MCAO group was increased with CT treatment. The protein level of FOXP3 and the phosphorylation of STAT5 were recovered in the CD4^+^CD25^+^ Treg cells of model group after treated with CT. Importantly, the effects of CT treatment were blocked by treatment with the inhibitor STAT5-IN-1 in CD4^+^ T cells of the MCAO model.

**Discussion and conclusion:**

Our findings not only enhance the understanding of the mechanisms underlying CT treatment, but also indicate its potential value as a promising agent in the treatment of ischaemic stroke. Further study will be valuable to examine the effects of CT on patients with ischaemic stroke.

## Introduction

Ischaemic stroke is a common acute cerebrovascular disease which leads to brain tissue damage and even disability (Aries et al. 2010). Most ischaemic strokes are caused by transient or permanent occlusion of an ischaemic blood vessel leading to the subsequent development of a brain infarction (Sommer 2017). The outcomes of the current standards of care for treatment of ischaemic stroke are still far from satisfactory. Therefore, novel effective therapeutic methods are needed.

Cryptotanshinone (CT) is a fat-soluble diterpene quinone compound that is derived from the dried roots and rhizomes of *Salvia miltiorrhiza* Bunge (Lamiaceae) (Yin et al. 2009). CT has multiple properties in the treatment of certain human diseases, including anticancer, anti-inflammatory, antioxidative, antidiabetic, and antiobesity properties (Kang et al. 2000; Hur et al. 2005; Huang et al. 2013; Li et al. 2015). Our previous findings indicated that CT may be a promising therapy for the treatment of cerebral stroke (Zhu et al. 2017). CT has been shown to suppress the growth of renal carcinoma cells through inhibition of the STAT3 signalling pathway (Chen et al. 2017) and has been shown to contribute to the treatment of liver cancer through decreasing the level of phosphorylated-STAT3 (Li et al. 2016). *Salvia miltiorrhiza* (Danshen) has been reported to attenuate cerebral ischaemic injury through the reduction of platelet activation in rats (Fei et al. 2017). Therefore, it is valuable to further examine the molecular mechanisms underlying CT protection in ischaemic stroke in rats.

Previous reports have suggested that inflammation plays an important role in the pathogenesis of ischaemic stroke (Pei et al. 2015; Shekhar et al. 2018). Regulatory T cells (Treg) are essential in maintaining the homeostasis of inflammation (Shimon et al. 2008) and natural Tregs that express CD4, CD25, and FOXP3 have been well characterised (Hori et al. 2017). Upregulation of CD4^+^CD25^+^FOXP3^+^ Tregs are beneficial in stroke-induced immunosuppression (Shimon et al. 2008). Tregs protect the brain against ischaemic injury via modulation of inflammatory processes and previous evidence suggests that TGF-β contributes to the production of CD4 ^+^ CD25 ^+^ FOXP3^+^ Tregs leading to protective effects in autoimmune diabetes (Godebu et al. 2008; Lu et al. 2010). However, the precise molecular mechanisms require further investigation.

Signal transducer and activator of transcription 5 (STAT5) is a transcription factor in multiple cell types, and is activated by certain cytokines (Isaksen et al. 1999). Previous studies have shown that STAT5 exhibits binding activity in T cells (Beadling et al. 1994). STAT5 is not only involved in the differentiation phase of Treg cells, but also induces the suppressive capabilities at mature phase and maintains the balance of FOXP3 in Treg cells (Owen and Farrar 2017). Therefore, STAT5 may be an effective target for the regulation of metabolism in Treg cells.

In the current study, we established the middle cerebral artery occlusion (MCAO) rat model and examined the effect of CT in this model. This research not only enhances the understanding of the molecular mechanisms underlying CT treatment but also indicate the potential value of CT in the treatment of ischaemic stroke.

## Materials and methods

### Development of the MCAO model in rats

All experiments were performed according to the National Institute of Health Guide for the Care and Use of Laboratory Animals and the ARRIVE guidelines. This analysis was approved by both the Institutional Animal Care and Use and the Ethics Committees of Jinhua Central Hospital, Zhejiang province.

Twenty male Sprague-Dawley rats (200 ± 20 g; Slarc, Shanghai, China) were randomly allocated into four groups (*n* = 5); the sham operation (NC), the MCAO model (model), the MCAO and the CT (15 mg/kg) treatment (Model + C15), and the MCAO and TGF-β (5 ng/mL) treatment group (Model + TGF-β). CT was purchased from Chengdu Best Biotechnology Co., Ltd (Sichuan, China).

The MCAO surgery was performed as described previously (Longa et al. 1989; Jing et al. 2010). Briefly, the rats were anaesthetised with 10% chloral hydrate (3 mL/kg) by intraperitoneal injection. The left common carotid artery and the external carotid artery were exposed and isolated through a ventral midline cervical incision. A 4-0 monofilament nylon suture (Beijing Sunbio Biotech Co Ltd, Beijing, China) with a rounded tip was introduced from the common carotid artery lumen into the internal carotid artery to occlude the origin of the left middle cerebral artery (MCA). The left MCA was occluded with the filament for 60 min and the filament was withdrawn to allow MCA reperfusion. In the sham-operated group, a left neck incision was made to expose the arteries but the nylon filament was not inserted into the internal carotid artery. All efforts were made to minimise animal suffering. Twenty-four h after reperfusion, rats were anaesthetised and sacrificed, and brain and blood were collected.

### Flow cytometry (FCM) assay

The blood samples were diluted in a PBS solution (1:1). Mononuclear cells were obtained by centrifugation (2000 rpm, 20 min) in a lymphocyte separation medium. The cell concentration was adjusted to 1 × 10^6^/mL and the cells were cultured with CT or the STAT5 inhibitor [STAT5-IN-1 (47 μM, 285986-31-4, Millipore, USA)] as indicated. Antibodies against CD4, CD25, and FOXP3 were added separately to the diluted cell solution, according to the instructions provided by the manufacturer (Ebioscience, San Diego, CA, USA). A FACS Calibur flow cytometer (Becton Dickinson, USA) was used for data collection. Data analysis was performed using the CellQuest software (Becton Dickinson, Bedford, MA, USA). Three replicates were performed for each sample.

### Histopathology assay

All sample tissues were fixed in 10% formalin for 48 h and subsequently embedded with paraffin (4 mm). The samples were then cut into slices (5 μm) using a microtome (Leike, China). A series of xylene baths and graded alcohols were utilised to deparaffinize and rehydrate the tissue. Haematoxylin and eosin (H & E) staining was performed for nuclear counterstaining after slices with reacted with diaminobenzidine (DAB) substrate. H & E stained sections were scored as described previously (Kitagawa et al. 1991). Three replicates were used for each sample.

### 2,3,5-Triphenyltetraolium chloride staining

Rats were sacrificed under deep anaesthesia and the skull and cervical vertebra were separated by using tissue scissors. The brain of the rat was carefully removed from the skull though the foramen magnum and the brain tissues were coronally sliced into 2 mm thick slices. The 2, 3, 5-triphenyltetrazolium chloride (TTC) staining assay was performed according as described by the manufacturer (Njjctech, Chian). The sections were analysed using ImageJ software (NIH, USA). The non-infarcted region appeared in red and the white region represents the infarct brain region. The percentage of infarct volume was assessed by (cerebral infarct volume/whole brain volume) × 100. Three replicates were performed for each sample. All efforts were made to minimise animal suffering.

### Western blots

Total protein lysates were isolated from the indicated cells using RIPA lysis buffer (JRDUN, Shanghai, China) with an EDTA-free protease inhibitor cocktail (Roche, Germany). An Enhanced BCA protein assay kit (Thermo Fisher, USA) was utilised to measure the protein concentration. A total of 25 μg protein was separated using a 10% SDS-PAGE gel and was transferred to a nitrocellulose membrane (Millipore, USA) overnight. The membranes were probed at 4 °C overnight with the primary antibodies: FOXP3 (1:1000), STAT5 (1:500), p-STAT5 (1:500) (Abcam, UK), and GAPDH (1:2000) (CST, USA) followed by incubation with the secondary antibody (antiserum-HRP tagged goat IgG anti-rabbit 1:1000; Beyotime, China) for 1 h at 37 °C. An enhanced chemiluminescence system (Tanon, China) was used to quantify the protein content. Each sample was tested in triplicate and GAPDH was used as the internal loading control.

### Determination of serum biochemical indicators

Blood samples collected from the abdominal aorta of rats were stored on ice prior to centrifugation at 2100 *g* for 10 min within 1 h of collection. The supernatant was collected for the determination of biochemical indicators. The levels of ALT, AST, BUN, and Cr were measured in the serum as sensitive indicators of liver damage. This was performed as described in the manufacturer's protocol of the diagnostic kits using a CHEMIX-180 automatic biochemistry analyser (Sysmex Corporation).

### Statistical analysis

Statistical analyses were performed using GraphPad Prism software Version 7.0 (CA, USA). Data were presented as the mean ± SD of at least three samples. Statistical significance was assessed using ANOVA for multiple comparisons. A *p*-value of < 0.05 was considered statistically significant.

## Results

### CT attenuates the infarct region in the MCAO model

**Figure 1.**
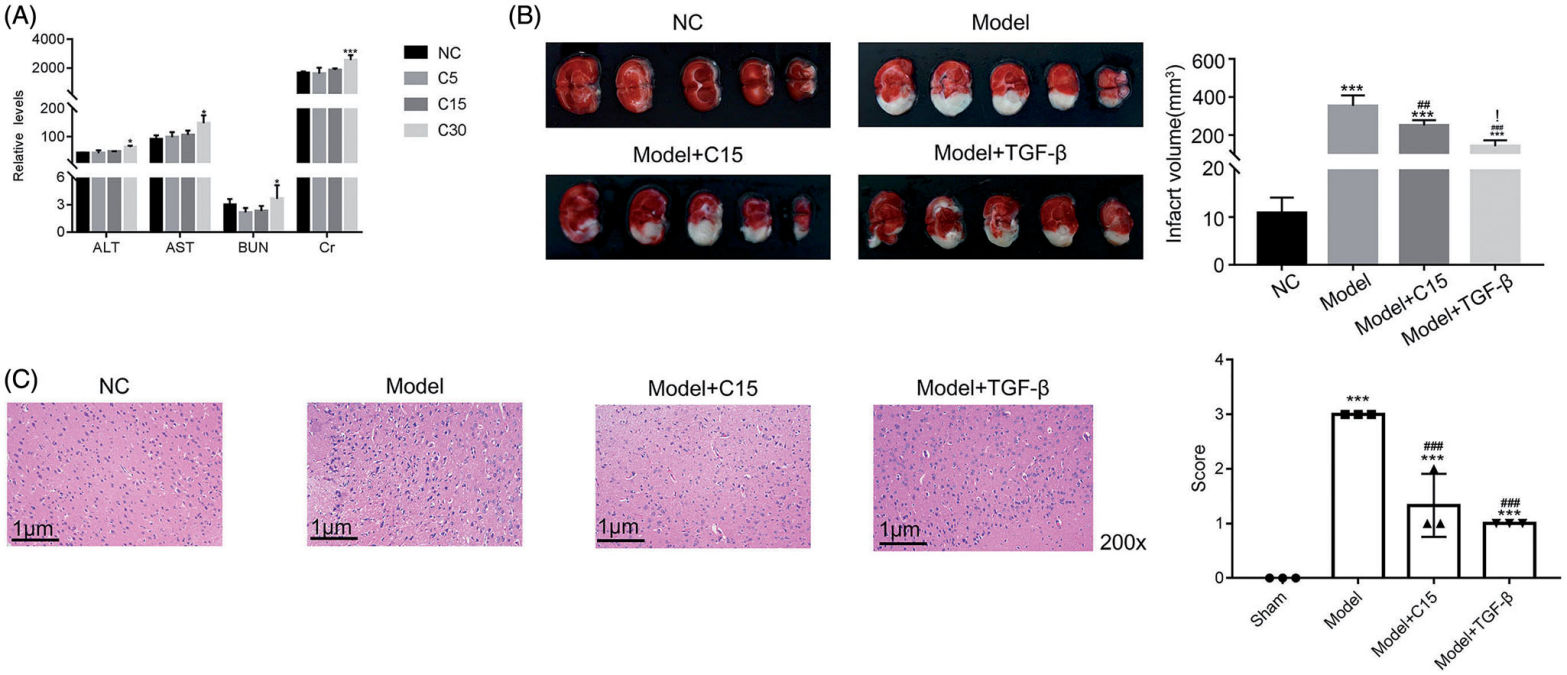
CT attenuates the infarct region in the MCAO model. (A) The serum levels of alanine transaminase (ALT), aspartate aminotransferase (AST), blood urea nitrogen (BUN), and creatinine (Cr) were assessed to evaluate hepatorenal function. * *p* < 0.05 vs. NC, *** *p* < 0.001 vs. NC. (B) The infarct region of rats subjected to MCAO and CT treatments were examined by the triphenyltetrazolium chloride (TTC)-stained assay. *** *p* < 0.001 vs. NC; ## *p* < 0.01 vs. Model, ### *p* < 0.001 vs. Model;! *p* < 0.05 vs. Model + C15 (C) Haematoxylin-eosin (HE) stained serial coronal brain sections from rats subjected to MCAO and CT treatments. Magnification, 200 ×. *** *p* < 0.001 vs. NC; ## *p* < 0.01 vs. NC; ### *p* < 0.001 vs. Model.

In order to determine the appropriate dose of CT, we tested the effect of CT on the hepatorenal function of the normal rat. The serum levels of alanine transaminase (ALT), aspartate aminotransferase (AST), blood urea nitrogen (BUN), and creatinine (Cr) were determined to evaluate hepatorenal function. As shown in Figure 1(A), the levels of ALT, AST, BUN, and Cr in the C5 or C15 treatment group were not significantly different than that of the control group. Our results demonstrate that a lower dose of CT treatment (5 and 15 mg/kg) did not affect the hepatorenal function of normal rat, while higher doses of CT treatment (30 mg/kg) may lead to hepatorenal dysfunction. Therefore, C30 may exhibit a toxic effect, so we chose to continue with 15 mg/kg of CT in subsequent experiments.

MCAO surgery was performed on rats under general anaesthesia and the sham surgery was performed to develop a negative control (NC). MCAO rats were treated with CT (15 mg/kg; Model + C15).

Previous report has demonstrated that TGF-β activation ameliorates cerebral ischemia/reperfusion injury after isoflurane post-conditioning in rats (Peng et al. 2019). In the present study, the recombinant protein TGF-β was used to treat model rat as positive control (5 ng/mL, Model + TGF-β).

The TTC assay was used to stain brain tissues. As shown in Figure 1(B), in the NC group, there was little infarcted region. Meanwhile, the MCAO operation led to significant infarction. Interestingly, the infarct region was smaller in the CT treatment group than in the model group. Similar results were also observed in the histopathology assay (Figure 1(C)). These findings indicate that the MCAO model was successfully induced. Our findings also indicated that treatment with CT also reduced the infarct region in the MCAO model. Interestingly, the recombinant protein TGF-β also presented a similar effect in the MCAO model.

#### CT-targeted FOXP3 and STAT5 in mononuclear cells of the MCAO model

**Figure 2.**
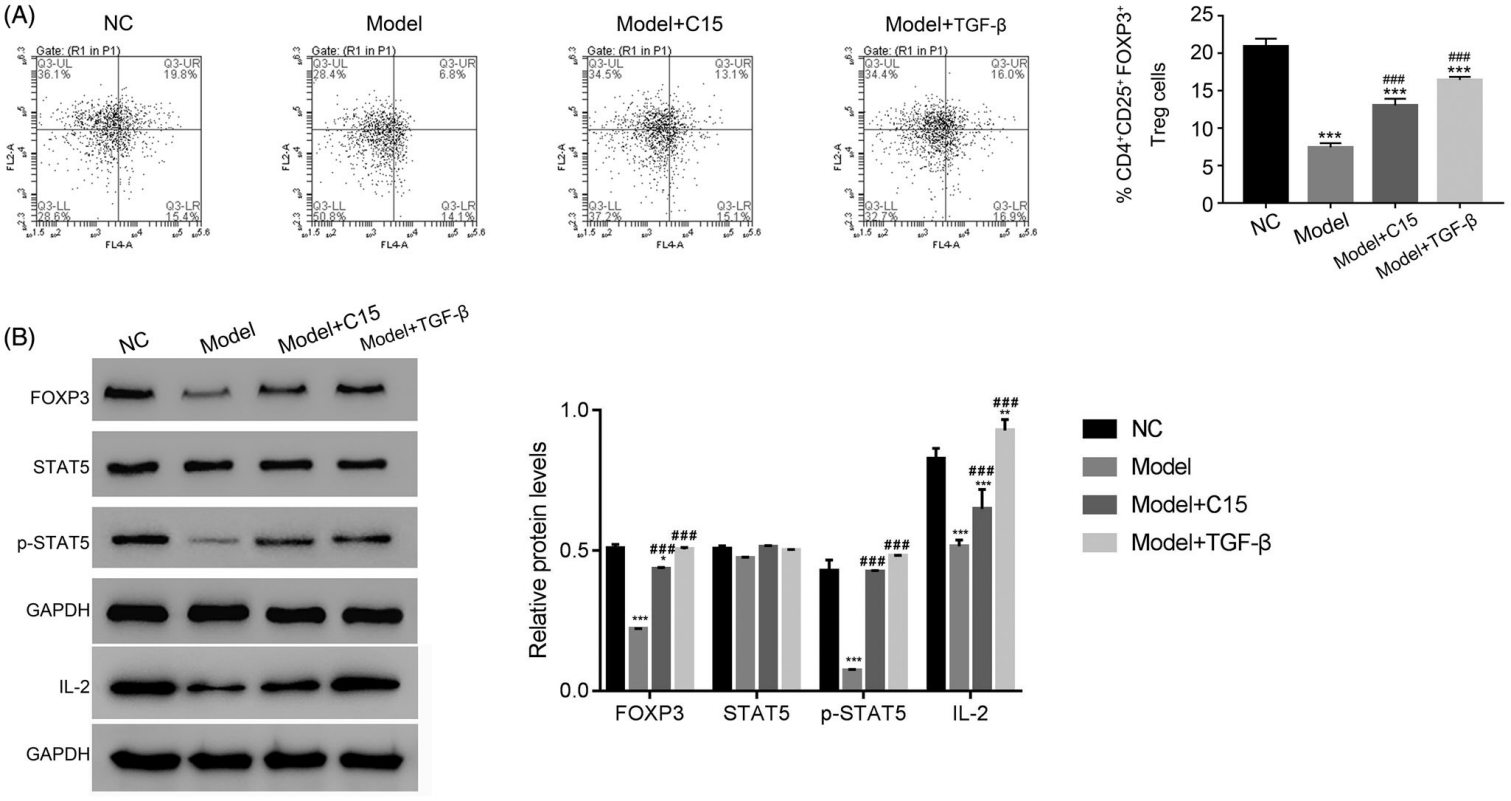
CT-targeted STAT5 in mononuclear cells of the MCAO model. (A) CT treatment promoted the number of CD25^+^FOXP3^+^ Treg cells in the mononuclear cells of the MCAO model. *** *p* < 0.001 vs. NC; ### *p* < 0.001 vs. Model. (B) Western blotting was used to examine the protein levels of FOXP3, STAT5, p-STAT5 and IL-2 in different mononuclear cells, as indicated. * *p* < 0.05 vs. NC, ** *p* < 0.01 vs. NC, *** *p* < 0.001 vs. NC; ### *p* < 0.001 vs. Model.

To further examine the effect of CT on the MCAO model, mononuclear cells were separated from peripheral blood of different groups of rats. Flow cytometry was used to analyse the percentage of CD4^+^CD25^+^FOXP3^+^ Treg cells in the different groups, as indicated. As presented in Figure 2(A), the percentage of CD4^+^CD25^+^FOXP3^+^ Treg cells was significantly reduced in the model group as compared with that of the NC group. However, CT treatment significantly increased the number of CD4^+^CD25^+^FOXP3^+^ Treg cells in the model group, suggesting the CT increases production of CD4^+^CD25^+^FOXP3^+^ Treg cells in the peripheral blood in the MCAO model. The production of CD4^+^CD25^+^FOXP3^+^ Treg cells was also increased in the presence of TGF-β.

We also separated the CD4^+^CD25^+^ Treg cells from the mononuclear cells in the different groups, as indicated, using magnetic beads. Western blotting was performed to quantify the protein levels of FOXP3, STAT5, and phosphorylated-STAT5 (p-STAT5) in the CD4^+^CD25^+^ Treg cells of the different groups, as indicated. The protein levels of FOXP3 were reduced in CD4^+^CD25^+^ Treg cells of the model group. However, the levels of FOXP3 were significantly induced by CT. Our result indicated that p-STAT5 in the CD4^+^CD25^+^ Treg population also increased after CT treatment in the model group (Figure 2(B)). Moreover, we also examined the protein levels of IL-2, the downstream regulator of STAT5 pathway. Clearly, the protein level of IL-2 was downregulated in model cells, while significantly upregulated after treated with CT. Together, these findings suggest that CT may target STAT5 in mononuclear cells of the MCAO model.

We also determined the phosphorylation of STAT3 in the model group. As shown in Figure S1, the phosphorylation of STAT3 was significantly reduced in the MCAO model compared with that in the sham group. However, the CT treatment (C15) did not significantly improve the phosphorylation of STAT3 in the MCAO group.

#### CT promoted the production of CD25^+^FOXP3^+^ Treg cells in CD4^+^ T cells in the MCAO model

**Figure 3.**
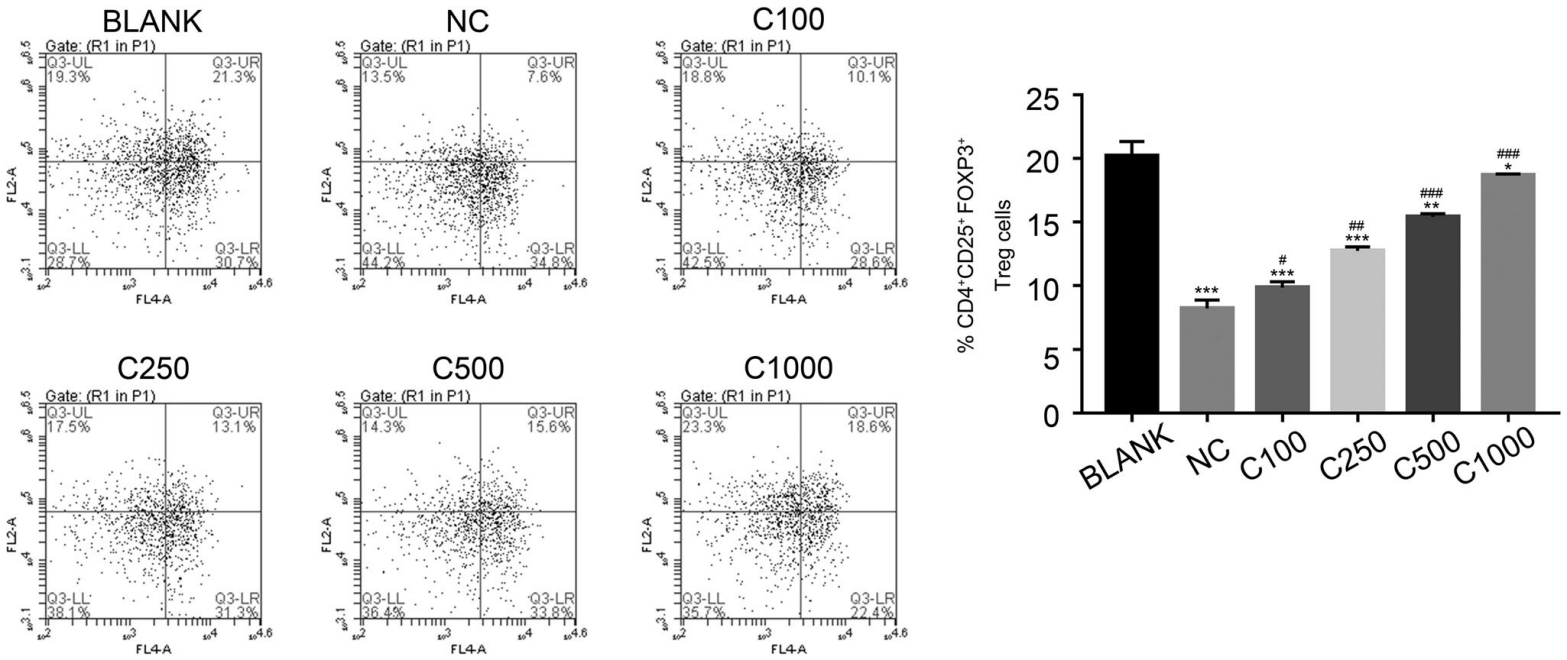
CT promotes the production of CD25^+^FOXP3^+^ Treg cells in CD4^+^ T cells of the MCAO model. * *p* < 0.05 vs. BLANK, ** *p* < 0.01 vs. BLANK, *** *p* < 0.001 vs. BLANK; # *p* < 0.05 vs. NC; ## *p* < 0.01 vs. NC; ### *p* < 0.001 vs. NC. C100, C250, C500 and C1000 means the cryptotanshinone in the concentration of 100, 250, 500 and 1000 µg/mL, respectively.

The CD4^+^ T cells in the MCAO model were separated from the peripheral blood of the different rat groups and cultured with varying concentrations of CT, including 100, 250, 500, and 1000 µg/mL. As shown in Figure 3, the percentage of CD4^+^CD25^+^FOXP3^+^ cells increased in a dose dependent manner with treatment of CT. These findings suggest that CT also promotes the production of CD25^+^FOXP3^+^ Treg cells in CD4^+^ T cells.

#### The effects of CT treatment are abolished by the STAT5 inhibitor STAT5-IN-1 in CD4^+^ T cells from the MCAO model

**Figure 4.**
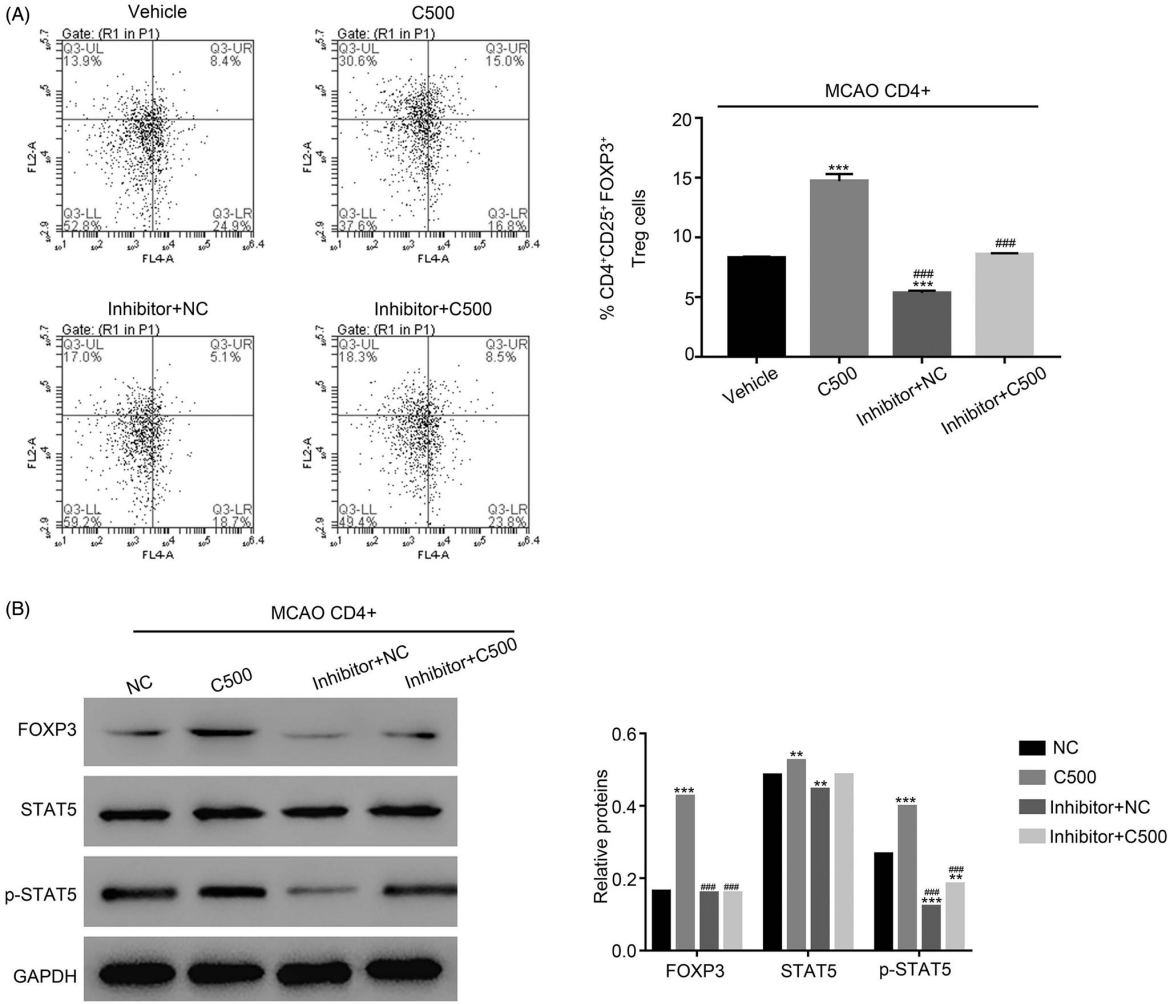
The effect of CT was abolished by the STAT5 inhibitor STAT5-IN-1 in. (A) The production of CD25^+^FOXP3^+^ cells was inhibited by treatment with the STAT5-IN-1 inhibitor in CD4^+^ T cells with the treatment of CT. *** *p* < 0.001 vs. NC; ### *p* < 0.001 vs. C500. (B) The protein levels of FOXP3 and p-STAT5 were suppressed by the inhibitor STAT5-IN-1 in CD4^+^ T cells with the treatment of CT. *** *p* < 0.001 vs. NC; ### *p* < 0.001 vs. C500.

To further examine the relationship between STAT5 and CT in CD4^+^ T cells of the MCAO group, a specific STAT5 inhibitor STAT5-IN-1 (47 μM) was used to culture CT (500 µg/mL) treated CD4^+^ T cells. As presented in Figure 4(A), the numbers of CD25^+^ FOXP3^+^ cells were remarkably increased in CD4^+^ T cells with the treatment of CT as compared with that of the NC cells. However, this effect was significantly inhibited by treatment with the STAT5 inhibitor STAT5-IN-1. Interestingly, the inhibitor STAT5-IN-1 also suppressed the expression of FOXP3 and phosphorylated-STAT5 in C500 cultured CD4^+^ T cells (Figure 4(B)). The effects of CT were remarkably abolished by the STAT5 inhibitor STAT5-IN-1 in CD4^+^T cells of MCAO model, suggesting that STAT5 is the potential target of CT in the MCAO model.

#### CT treatment improved the phosphorylation of STAT5 at Tyr694 site in the MCAO model

**Figure 5.**
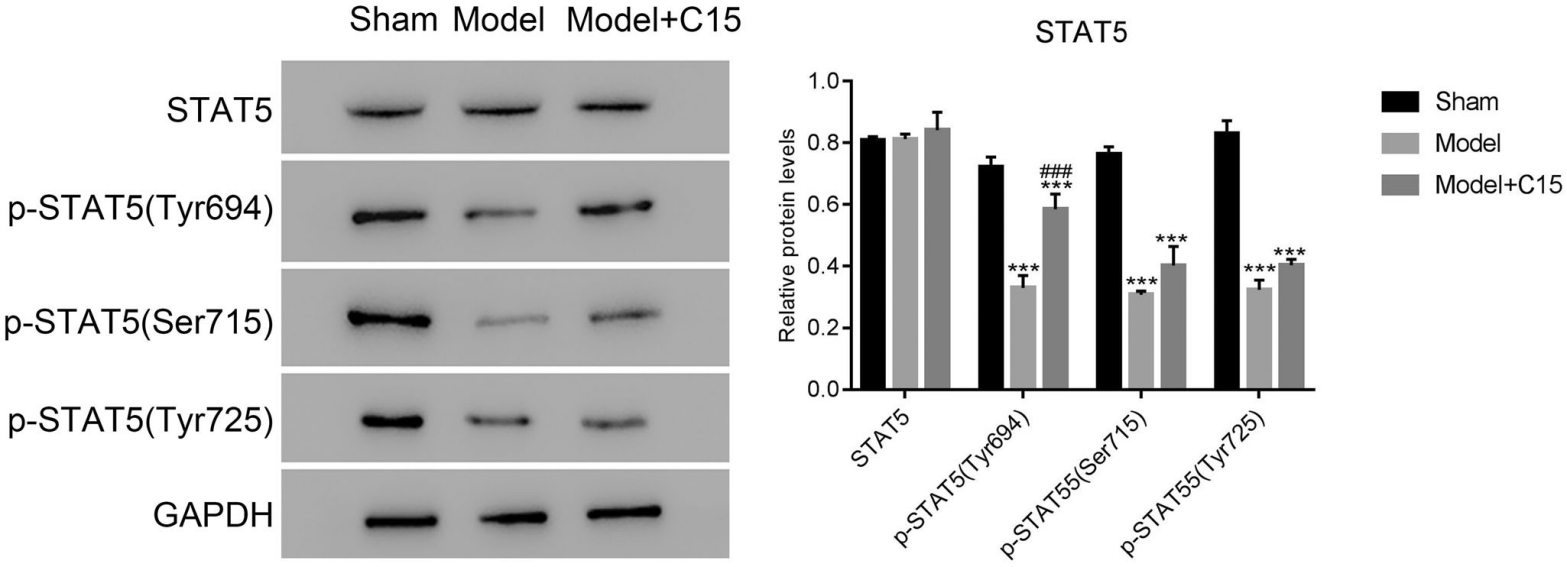
CT treatment increased levels of phosphorylated STAT5 at Tyr694 in the MCAO model. ***p < 0.001 vs. Sham; ###*p* < 0.001 vs. Model.

To further explore the relationship between CT and STAT5, we examined the phosphorylation of STAT5 at three different sites with CT treatment, including Tyr694, Ser715, and Tyr725. As presented in Figure 5, the phosphorylation of STAT5 at these three different sites was also downregulated in MCAO model. After CT treatment, the phosphorylation of STAT5 showed no significant differences at Ser715 and Tyr725. Our results suggest that the phosphorylation of STAT5 at the Tyr694 site significantly increased with CT treatment in the model group. Therefore, CT treatment improved the phosphorylation of STAT5 mainly at the Tyr694 site in MCAO model.

## Discussion

Ischaemic stroke is one of the most common diseases that threatens human health and quality of life. Although the present therapies have contributed to the treatment of ischaemic stroke, the majority of patients still suffer from varying degrees of symptoms from ischaemic infarction (Keun-Sik and Saver 2010). Therefore, an effective agent for the treatment of ischaemic infarction is needed.

In the present study, we examined the function of CT in ischaemic stroke using a rat MCAO model. Our analysis demonstrated that CT treatment reduces the infarct region in the MCAO model. These results indicate that CT is a promising agent for the treatment for ischaemic infarction.

Regulatory T cells (Tregs) play a critical role in the maintenance of immune homeostasis and the suppression of redundant immune responses (Battaglia et al. 2006). Previous reports have demonstrated that depletion of circulating Tregs increased brain damage in the MCAO model (Liesz et al. 2009; Satoru et al. 2009). Therefore, maintaining the balance of Treg metabolism is essential for stroke treatment.CD4^+^CD25^+^FOXP3^+^ Treg cells inhibit multiple physiological and pathological immune responses (Chen et al. 2013). Stroke often induces the dysfunction of the peripheral immune system (Halina et al. 2006; McCombe and Read 2008; Emsley and Hedley 2010). In this study, our results suggest that CT treatment improved the production of CD4^+^CD25^+^FOXP3^+^ Treg cells in the peripheral blood of the MCAO model. Moreover, the protein levels of FOXP3 were promoted by CT in CD4^+^T cells of the MCAO model. CT may provide benefit to those suffering from ischaemic stroke through increasing the production of CD4^+^CD25^+^FOXP3^+^ Treg cells.

In the current study, we also examined the effect of CT on rat hepatorenal function. Our results indicated that CT treatment (30 mg/kg) may lead to the dysregulation of rat hepatorenal function, suggesting that the higher dose of CT may have toxic effects on hepatorenal function.

Previous reports have indicated that CT alleviates cardiac fibrosis in type 1-like diabetic rats through regulation of the activity of STAT3 (Lo et al. 2017). Moreover, a similar mechanism has also been obtained in rheumatoid arthritis analysis (Wang et al. 2017). CT has also been identified as a dual inhibitor of p-STAT5 and p-STAT3 in chronic myeloid leukaemia (Dong et al. 2018). In the present study, our results indicate that the phosphorylation of STAT5 (at Tyr694) was significantly upregulated by CT in CD4^+^ T cells of the MCAO model. The effect of CT was abolished by the STAT5 inhibitor STAT5-IN-1. Taken together, our results demonstrate that CT possesses therapeutic effects on ischaemic stroke through improving the phosphorylation of STAT5. Therefore, the phosphorylation of STAT5 might present a diverse function in human diseases. Although our findings differ from a previous report, they illustrate the critical role of CT in ischaemic stroke treatment. Future studies are needed to further explore the mechanisms of CT regulation of STAT5 in ischaemic stroke.

## Conclusions

We analysed the effects of CT on the MCAO rat model. Our results further enhanced the understanding of the molecular mechanisms underlying CT treatment and indicate its potential value as a promising agent in the treatment of ischaemic stroke.

## Supplementary Material

Supplemental Material

Supplemental Material
