# Supplementary material for: Cryptotanshinone possesses therapeutic effects on ischaemic stroke through regulating STAT5 in a rat model
Source: Pharm Biol. 2021 Apr 29;59(1):465–71. doi: 10.1080/13880209.2021.1914672 (PMC8871624; doi:10.1080/13880209.2021.1914672)
Supplement: Supplemental Material [file IPHB_A_1914672_SM1523.docx]

Figure S1: CT treatment did not affect the phosphorylation of STAT3 in the MCAO model. *** *p* < 0.001 vs. sham.
