# Supplementary figures and images for: Cryptotanshinone possesses therapeutic effects on ischaemic stroke through regulating STAT5 in a rat model
Source: Pharm Biol. 2021 Apr 29;59(1):465–71. doi: 10.1080/13880209.2021.1914672 (PMC8871624; doi:10.1080/13880209.2021.1914672)

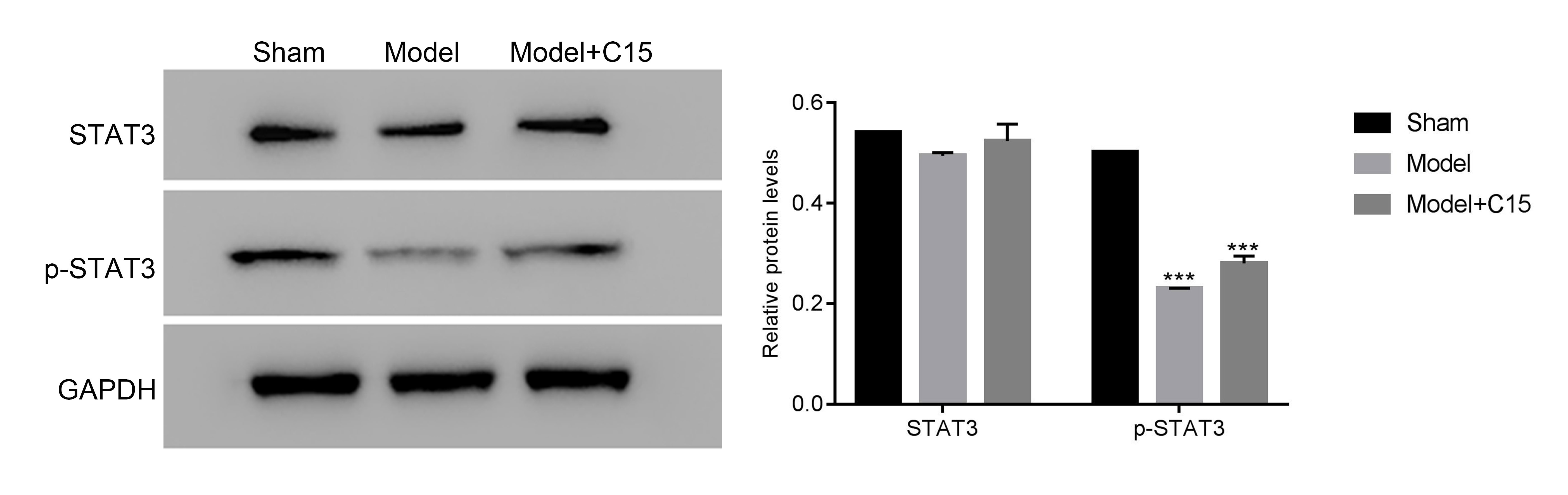

Supplement: Supplemental Material [file IPHB_A_1914672_SM1431.jpg]
